# Emergency Department Utilization and Outpatient Care Continuity Among White and Hispanic Patients With Inflammatory Bowel Disease at a County Health System

**DOI:** 10.7759/cureus.102071

**Published:** 2026-01-22

**Authors:** Matthew Orosa, Andrew Chang

**Affiliations:** 1 Internal Medicine, Riverside University Health System Medical Center, Moreno Valley, USA; 2 Gastroenterology and Hepatology, Loma Linda University Medical Center, Loma Linda, USA

**Keywords:** emergency department utilization, health disparities, hispanic patients, inflammatory bowel disease, safety net health system

## Abstract

Introduction

Inflammatory bowel disease (IBD), including Crohn’s disease and ulcerative colitis, is associated with emergency department (ED) utilization and variable engagement with outpatient gastroenterology (GI) care. We examined ED utilization and outpatient GI care engagement among White and Hispanic patients with IBD within a county health system.

Methods

We conducted a retrospective cohort study of adult patients (≥18 years) with IBD receiving care within a single county health system between January 1, 2016, and December 31, 2024. Patients self-identifying as non-Hispanic White or Hispanic were included. Primary outcomes included the unadjusted count of IBD-related ED visits per patient during observed follow-up. Secondary outcomes included the presence of any ED visit, missed outpatient GI appointments, and GI follow-up within 90 days after an index ED visit among patients with at least one IBD-related ED visit and sufficient follow-up time. Comparisons were performed using chi-square testing for categorical variables and the Wilcoxon rank-sum test for continuous variables.

Results

Among 138 included patients, Hispanic patients experienced a significantly higher median number of IBD-related ED visits compared with White patients (P = 0.008), while the proportion of patients with at least one ED visit did not differ by race/ethnicity. Rates of missed GI appointments and outpatient GI follow-up within 90 days after ED encounters were similar between groups.

Conclusions

Within this county health system, Hispanic patients with IBD experienced more frequent ED utilization than White patients, without differences in outpatient GI follow-up following ED encounters.

## Introduction

Inflammatory bowel disease (IBD), encompassing Crohn’s disease and ulcerative colitis, is a chronic condition associated with recurrent disease flares, complications, and high healthcare utilization, particularly emergency department (ED) visits [1]. Prior studies have demonstrated significant racial and ethnic disparities in IBD-related healthcare utilization, including differences in ED visits, hospitalizations, and access to specialty care [1,2].

National analyses have shown that racial and ethnic minority populations with IBD may experience barriers to timely outpatient gastroenterology (GI) care, leading to greater reliance on acute care services [2]. Hispanic patients with IBD have been shown to differ in sociodemographic characteristics, disease experiences, and health-related quality of life compared with non-Hispanic White patients [3].

Despite growing recognition of these disparities, data examining ED utilization and outpatient care continuity among Hispanic patients with IBD within safety-net or county health systems remain limited. Understanding whether disparities persist following acute care encounters is essential for informing targeted interventions. This study aimed to compare ED utilization patterns and outpatient GI care engagement between White and Hispanic patients with IBD within a county health system.

## Materials and methods

Study design and population

We conducted a retrospective observational cohort study using electronic medical records from a single county-based health system. Adult patients (≥18 years) with Crohn’s disease or ulcerative colitis were identified based on the presence of at least two IBD-related diagnostic codes and supporting documentation of IBD in outpatient GI clinic notes.

Patients were required to have at least 12 months of follow-up within the health system and self-identified race/ethnicity as non-Hispanic White or Hispanic. Patients with indeterminate colitis, missing race/ethnicity data, or primary GI care outside the system were excluded.

Outcomes

The primary outcome was the unadjusted count of IBD-related ED visits per patient during observed follow-up. Because all patients were required to have at least 12 months of follow-up within the health system, visit counts were used as a descriptive measure rather than person-time-adjusted rates. Secondary outcomes included the presence of at least one IBD-related ED visit, missed or cancelled outpatient GI appointments, and outpatient GI follow-up within 90 days after an index ED visit.

Missed outpatient GI appointments were defined as scheduled clinic visits that were not completed, including patient no-shows or cancellations, as recorded in the electronic scheduling system. Clinic-initiated cancellations and rescheduled appointments were not analyzed separately due to limitations of the administrative data structure. Analyses of post-ED GI follow-up were restricted to patients with at least one IBD-related ED visit and a minimum of 90 days of follow-up after the index ED encounter; ED encounters occurring within 90 days of the study end date were excluded to ensure adequate follow-up time. The index ED visit was defined as the first IBD-related ED encounter during the study period.

IBD-related ED visits were identified using diagnostic codes for Crohn’s disease and ulcerative colitis documented in the electronic medical record within the county health system. ED encounters were classified as IBD-related when an IBD diagnosis code was present for the visit. Coding was based on routinely collected administrative data and was not supplemented by formal chart-level validation. Accordingly, outcomes reflect health system-level utilization patterns rather than adjudicated clinical events.

Statistical analysis

Categorical outcomes were compared using Fisher’s exact testing due to small cell sizes in several analyses and differing analytic denominators across outcomes. Continuous variables were compared using the Wilcoxon rank-sum test. All P-values were two-sided, and a P-value < 0.05 was considered statistically significant.

## Results

Baseline characteristics

A total of 160 patients with IBD were initially identified for review. After applying the inclusion and exclusion criteria, 138 patients were included in the final analytic cohort, comprising 75 White patients and 63 Hispanic patients. Hispanic patients were younger at baseline compared with White patients and were more likely to carry a diagnosis of ulcerative colitis rather than Crohn’s disease. Medicaid insurance coverage was highly prevalent in both racial/ethnic groups, although a greater proportion of Hispanic patients were insured through Medicaid. Baseline demographic and clinical characteristics of the study population are summarized in Table 1.

**Table 1:** Baseline Characteristics of White and Hispanic Patients With Inflammatory Bowel Disease

Characteristic	White (n = 75)	Hispanic (n = 63)
Age, median (years)	50	43
Female sex, n (%)	24 (32.0%)	21 (33.3%)
IBD type		
Crohn’s disease, n (%)	14 (18.7%)	1 (1.6%)
Ulcerative colitis, n (%)	61 (81.3%)	62 (98.4%)
Insurance		
Medicaid, n (%)	60 (80.0%)	55 (87.3%)
Other (Medicare, private, self-pay), n (%)	15 (20.0%)	8 (12.7%)

ED utilization

Overall, the proportion of patients experiencing at least one IBD-related ED visit did not differ significantly between White and Hispanic patients. In contrast, among patients who utilized emergency care, Hispanic patients had a significantly higher median number of IBD-related ED visits compared with White patients (P = 0.008). This finding suggests a greater frequency of recurrent acute care utilization among Hispanic patients. These results are consistent with prior studies demonstrating racial and ethnic disparities in ED utilization among individuals with IBD [1,4].

Outpatient GI engagement

Engagement with outpatient GI care was similar between racial/ethnic groups. Specifically, rates of missed outpatient GI appointments did not differ significantly between Hispanic and White patients. In addition, the proportion of patients who completed a GI follow-up visit within 90 days of an IBD-related ED encounter was comparable across groups. Collectively, these findings suggest that disparities in ED utilization were not explained by differences in outpatient GI follow-up or appointment adherence following acute care encounters. ED utilization patterns and outpatient GI engagement by race/ethnicity are summarized in Table 2.

**Table 2:** Emergency Department Utilization and Outpatient Gastroenterology Engagement by Race/Ethnicity ^†^ Analysis restricted to patients with ≥1 IBD-related ED visit and ≥90 days of follow-up. IBD: Inflammatory bowel disease; ED: Emergency department; GI: Gastroenterology

Characteristic	White (n = 75)	Hispanic (n = 63)	P-value
≥1 IBD-related ED visit, n (%)	45 (60.0%)	44 (69.8%)	0.29
Number of IBD-related ED visits, median (range)	1.0 (0–3.0)	2.0 (0–5.0)	0.008
History of missed GI appointment, n (%)	15 (20.0%)	20 (31.7%)	0.05
GI follow-up within 90 days after ED visit, n (%)^†^	29 (64.4%)	22 (50.0%)	0.08

## Discussion

In this retrospective cohort study conducted within a county health system, Hispanic patients with IBD experienced more frequent ED utilization compared with White patients. Importantly, this difference was driven by a higher number of ED visits per patient rather than a higher likelihood of any ED presentation.

Prior studies have demonstrated disparities in IBD-related healthcare utilization across racial and ethnic groups, including increased ED use and reduced access to specialty care among minority populations [1,2,4]. Our findings extend this literature by demonstrating that higher ED utilization among Hispanic patients persists even within a safety-net system designed to provide care regardless of insurance status.

Despite differences in ED utilization frequency, outpatient GI follow-up after ED visits did not differ between Hispanic and White patients. This suggests that disparities in acute care use may arise upstream of outpatient care continuity, potentially related to disease burden, symptom severity, or social determinants of health, such as language barriers, health literacy, and transportation challenges [5-7].

Large database analyses have similarly demonstrated that Hispanic patients with IBD experience distinct patterns of healthcare utilization and treatment compared with White patients, underscoring the importance of addressing structural and social factors contributing to acute care reliance [5,8].

Limitations

This study is limited by its retrospective design, modest sample size, and single-center setting, which may limit generalizability. Disease severity and social determinants of health were not uniformly captured and could not be directly assessed.

In addition, comparisons were unadjusted and may be influenced by baseline differences in disease phenotype, including the predominance of ulcerative colitis among Hispanic patients. ED utilization was assessed using unadjusted visit counts rather than person-time-adjusted rates, and follow-up duration may have varied between individuals despite a minimum follow-up requirement. Finally, outcome definitions were based on administrative scheduling and diagnostic data, which may not fully capture clinical nuance.

## Conclusions

Within a county health system, Hispanic patients with IBD experienced a higher frequency of ED utilization compared with White patients, without differences in outpatient GI follow-up following ED encounters. These findings highlight the need for interventions addressing upstream drivers of acute care utilization among Hispanic patients with IBD.
